# Supplementary material for: MetaRibo-Seq measures translation in microbiomes
Source: Nat Commun. 2020 Jun 29;11:3268. doi: 10.1038/s41467-020-17081-z (PMC7324362; doi:10.1038/s41467-020-17081-z)
Supplement: Supplementary file 10 — Supplementary Data 7 [file 41467_2020_17081_MOESM10_ESM.zip › File2/Confidence_High_Taxonomy/29774_out.krona.html]

Javascript must be enabled to view this page.

members
magnitude
magnitudeUnassigned
count
unassigned
taxon
rank

29774\_out

429

2
superkingdom
429

phylum
976
429

class
200643
429

429
171549
order

428
815
family

genus
17
816

SRS013800\_contig\_number\_27125SRS015264\_contig\_number\_20495SRS015579\_contig\_number\_contig-100\_3412.138737SRS015890\_contig\_number\_17041SRS017191\_contig\_number\_17136SRS019068\_contig\_number\_101607SRS022137\_contig\_number\_contig-100\_7831.106362SRS023346\_contig\_number\_contig-100\_2521.58505SRS050925\_contig\_number\_35290SRS058145\_contig\_number\_contig-100\_13110.13111SRS058770\_contig\_number\_28236SRS075341\_contig\_number\_5201SRS097889\_contig\_number\_29446SRS101441\_contig\_number\_contig-100\_17133.17133SRS143466\_contig\_number\_contig-100\_16964.141784SRS143598\_contig\_number\_19265SRS148319\_contig\_number\_contig-100\_3392.104207
428

29
species

SRS011134\_contig\_number\_38234SRS012273\_contig\_number\_49046SRS013215\_contig\_number\_2334SRS013940\_contig\_number\_19924SRS016018\_contig\_number\_11038SRS018049\_contig\_number\_1298SRS018575\_contig\_number\_8339SRS024132\_contig\_number\_13867SRS024549\_contig\_number\_contig-100\_258.48737SRS024663\_contig\_number\_4724SRS042628\_contig\_number\_37645SRS043001\_contig\_number\_10144SRS043768\_contig\_number\_contig-100\_330.197489SRS045195\_contig\_number\_contig-100\_616.66181SRS045713\_contig\_number\_18361SRS046717\_contig\_number\_11520SRS052078\_contig\_number\_2143SRS056519\_contig\_number\_285SRS077024\_contig\_number\_1922SRS078419\_contig\_number\_34876SRS098717\_contig\_number\_23166SRS101268\_contig\_number\_320SRS1041145\_contig\_number\_36820SRS104636\_contig\_number\_25474SRS1055034\_contig\_number\_9082SRS142712\_contig\_number\_1051SRS147139\_contig\_number\_9423SRS148874\_contig\_number\_10863SRS149879\_contig\_number\_contig-100\_94.159265
357276


SRS011084\_contig\_number\_31614SRS011239\_contig\_number\_2374SRS011302\_contig\_number\_29287SRS011405\_contig\_number\_11119SRS012849\_contig\_number\_contig-100\_4317.179980SRS012902\_contig\_number\_955SRS012969\_contig\_number\_27395SRS013098\_contig\_number\_13513SRS013158\_contig\_number\_920SRS013382\_contig\_number\_346SRS013638\_contig\_number\_13978SRS013687\_contig\_number\_34879SRS013951\_contig\_number\_41288SRS013965\_contig\_number\_18280SRS014313\_contig\_number\_426SRS014412\_contig\_number\_19840SRS014459\_contig\_number\_22071SRS014534\_contig\_number\_869SRS014683\_contig\_number\_6443SRS014736\_contig\_number\_3486SRS014855\_contig\_number\_9655SRS014948\_contig\_number\_17371SRS014979\_contig\_number\_36645SRS015065\_contig\_number\_30599SRS015190\_contig\_number\_6913SRS015217\_contig\_number\_contig-100\_1096.174774SRS015431\_contig\_number\_32517SRS015486\_contig\_number\_622SRS015578\_contig\_number\_2503SRS015663\_contig\_number\_32433SRS015694\_contig\_number\_21185SRS015782\_contig\_number\_34778SRS015854\_contig\_number\_6573SRS015960\_contig\_number\_contig-100\_1857.133741SRS016056\_contig\_number\_1349SRS016095\_contig\_number\_26478SRS016203\_contig\_number\_11186SRS016335\_contig\_number\_45426SRS016381\_contig\_number\_contig-100\_610.30215SRS016495\_contig\_number\_19117SRS016629\_contig\_number\_contig-100\_3630.31499SRS016753\_contig\_number\_7182SRS016840\_contig\_number\_contig-100\_398.33777SRS016897\_contig\_number\_609SRS016954\_contig\_number\_18129SRS016989\_contig\_number\_11248SRS017103\_contig\_number\_38688SRS017247\_contig\_number\_6780SRS017307\_contig\_number\_5582SRS017375\_contig\_number\_615SRS017433\_contig\_number\_14437SRS017521\_contig\_number\_10883SRS017701\_contig\_number\_2930SRS017745\_contig\_number\_791SRS017821\_contig\_number\_32315SRS017859\_contig\_number\_5744SRS018133\_contig\_number\_12940SRS018351\_contig\_number\_2759SRS018427\_contig\_number\_4673SRS018541\_contig\_number\_11738SRS018623\_contig\_number\_13521SRS018817\_contig\_number\_11150SRS018836\_contig\_number\_2231SRS018888\_contig\_number\_contig-100\_2222.51778SRS018936\_contig\_number\_11333SRS019030\_contig\_number\_12642SRS019068\_contig\_number\_53438SRS019161\_contig\_number\_22173SRS019178\_contig\_number\_1506SRS019267\_contig\_number\_2035SRS019286\_contig\_number\_contig-100\_575.49708SRS019381\_contig\_number\_1037SRS019397\_contig\_number\_2743SRS019496\_contig\_number\_4796SRS019601\_contig\_number\_12091SRS019693\_contig\_number\_1444SRS019808\_contig\_number\_18173SRS019910\_contig\_number\_24709SRS019968\_contig\_number\_contig-100\_487.104481SRS020233\_contig\_number\_21686SRS020271\_contig\_number\_contig-100\_12985.12986SRS020328\_contig\_number\_31351SRS020394\_contig\_number\_contig-100\_983.106584SRS020508\_contig\_number\_164SRS020622\_contig\_number\_6356SRS020869\_contig\_number\_36749SRS021153\_contig\_number\_1593SRS021219\_contig\_number\_2745SRS021484\_contig\_number\_26036SRS021948\_contig\_number\_29857SRS022071\_contig\_number\_36011SRS022524\_contig\_number\_16660SRS022609\_contig\_number\_16097SRS022713\_contig\_number\_1031SRS023526\_contig\_number\_12155SRS023583\_contig\_number\_15054SRS023715\_contig\_number\_28090SRS023914\_contig\_number\_17521SRS023971\_contig\_number\_4020SRS024009\_contig\_number\_8158SRS024075\_contig\_number\_2955SRS024331\_contig\_number\_12978SRS024435\_contig\_number\_42971SRS024492\_contig\_number\_16971SRS024625\_contig\_number\_6754SRS042284\_contig\_number\_contig-100\_649.48623SRS043411\_contig\_number\_973SRS043667\_contig\_number\_5470SRS043701\_contig\_number\_12837SRS044535\_contig\_number\_13865SRS045004\_contig\_number\_1445SRS045244\_contig\_number\_7532SRS045739\_contig\_number\_506SRS045826\_contig\_number\_22107SRS046068\_contig\_number\_2881SRS046369\_contig\_number\_16485SRS046502\_contig\_number\_contig-100\_177.88398SRS046810\_contig\_number\_contig-100\_29.30SRS047044\_contig\_number\_1018SRS047433\_contig\_number\_19956SRS048164\_contig\_number\_7831SRS049402\_contig\_number\_9894SRS049446\_contig\_number\_3462SRS049712\_contig\_number\_11999SRS049773\_contig\_number\_15118SRS049896\_contig\_number\_22287SRS049995\_contig\_number\_36199SRS050026\_contig\_number\_contig-100\_2122.59019SRS050299\_contig\_number\_8583SRS050422\_contig\_number\_10554SRS050520\_contig\_number\_172SRS050752\_contig\_number\_11465SRS050801\_contig\_number\_contig-100\_1021.29068SRS050941\_contig\_number\_17697SRS050974\_contig\_number\_1327SRS050998\_contig\_number\_4848SRS051031\_contig\_number\_29873SRS051610\_contig\_number\_10887SRS051882\_contig\_number\_1004SRS052576\_contig\_number\_71SRS052697\_contig\_number\_17862SRS053087\_contig\_number\_2730SRS053214\_contig\_number\_868SRS053335\_contig\_number\_contig-100\_1257.105736SRS053356\_contig\_number\_contig-100\_254.275220SRS053398\_contig\_number\_12051SRS053649\_contig\_number\_912SRS054059\_contig\_number\_contig-100\_302.49005SRS054352\_contig\_number\_11678SRS054905\_contig\_number\_8774SRS055017\_contig\_number\_contig-100\_703.201883SRS055966\_contig\_number\_165SRS055982\_contig\_number\_11406SRS056259\_contig\_number\_30000SRS056273\_contig\_number\_24317SRS056537\_contig\_number\_52525SRS057478\_contig\_number\_1148SRS057480\_contig\_number\_10774SRS057717\_contig\_number\_2459SRS058070\_contig\_number\_4011SRS058268\_contig\_number\_contig-100\_15.16SRS058723\_contig\_number\_16SRS062427\_contig\_number\_8089SRS062654\_contig\_number\_21850SRS062701\_contig\_number\_7683SRS063127\_contig\_number\_167SRS063190\_contig\_number\_contig-100\_631.85008SRS063370\_contig\_number\_1869SRS063489\_contig\_number\_contig-100\_162.52016SRS063518\_contig\_number\_34197SRS063813\_contig\_number\_3336SRS063928\_contig\_number\_1381SRS063985\_contig\_number\_23446SRS064557\_contig\_number\_1102SRS064645\_contig\_number\_1449SRS064757\_contig\_number\_21603SRS064973\_contig\_number\_17SRS065176\_contig\_number\_14942SRS065397\_contig\_number\_1525SRS074586\_contig\_number\_50SRS074670\_contig\_number\_6030SRS074964\_contig\_number\_307SRS075078\_contig\_number\_21426SRS075398\_contig\_number\_2041SRS075716\_contig\_number\_3932SRS075773\_contig\_number\_35079SRS075821\_contig\_number\_15461SRS075878\_contig\_number\_20243SRS075984\_contig\_number\_13412SRS076756\_contig\_number\_18118SRS076876\_contig\_number\_196SRS076976\_contig\_number\_19019SRS077086\_contig\_number\_11504SRS077127\_contig\_number\_15408SRS077194\_contig\_number\_10323SRS077294\_contig\_number\_4251SRS077335\_contig\_number\_377SRS077392\_contig\_number\_27765SRS077454\_contig\_number\_17981SRS077502\_contig\_number\_2411SRS077589\_contig\_number\_1015SRS077641\_contig\_number\_15922SRS077730\_contig\_number\_2102SRS077849\_contig\_number\_22359SRS077991\_contig\_number\_110SRS078176\_contig\_number\_contig-100\_1007.139068SRS078242\_contig\_number\_29959SRS078665\_contig\_number\_3814SRS097920\_contig\_number\_29573SRS097958\_contig\_number\_2129SRS098061\_contig\_number\_12715SRS098571\_contig\_number\_15229SRS098644\_contig\_number\_14706SRS098827\_contig\_number\_1222SRS098881\_contig\_number\_10071SRS100907\_contig\_number\_4410SRS101376\_contig\_number\_18780SRS101433\_contig\_number\_7870SRS103987\_contig\_number\_740SRS104036\_contig\_number\_contig-100\_2366.55736SRS104084\_contig\_number\_1260SRS1041031\_contig\_number\_21769SRS1041032\_contig\_number\_17213SRS1041036\_contig\_number\_1448SRS1041037\_contig\_number\_15168SRS1041039\_contig\_number\_25895SRS1041090\_contig\_number\_1795SRS1041091\_contig\_number\_12298SRS1041092\_contig\_number\_8663SRS1041095\_contig\_number\_837SRS1041112\_contig\_number\_1084SRS1041116\_contig\_number\_1453SRS1041118\_contig\_number\_1027SRS1041129\_contig\_number\_11226SRS1041130\_contig\_number\_6060SRS1041133\_contig\_number\_10644SRS1041134\_contig\_number\_13226SRS1041136\_contig\_number\_26157SRS1041137\_contig\_number\_30093SRS1041138\_contig\_number\_9934SRS1041139\_contig\_number\_935SRS1041140\_contig\_number\_18132SRS1041141\_contig\_number\_14920SRS1041142\_contig\_number\_3343SRS1041143\_contig\_number\_6540SRS1041144\_contig\_number\_1627SRS1041147\_contig\_number\_4594SRS1041157\_contig\_number\_5735SRS1041164\_contig\_number\_888SRS104327\_contig\_number\_2060SRS104400\_contig\_number\_43561SRS104485\_contig\_number\_261SRS104693\_contig\_number\_4766SRS104912\_contig\_number\_18217SRS104975\_contig\_number\_22805SRS105082\_contig\_number\_17725SRS105153\_contig\_number\_2530SRS1054716\_contig\_number\_11780SRS1054928\_contig\_number\_3866SRS1055022\_contig\_number\_13169SRS1055038\_contig\_number\_5506SRS1055049\_contig\_number\_3663SRS1055056\_contig\_number\_9073SRS1055069\_contig\_number\_5100SRS1055076\_contig\_number\_10600SRS1055083\_contig\_number\_2627SRS1055095\_contig\_number\_1333SRS140492\_contig\_number\_30487SRS140513\_contig\_number\_12041SRS140645\_contig\_number\_contig-100\_565.26362SRS142503\_contig\_number\_4968SRS142542\_contig\_number\_11819SRS142599\_contig\_number\_14959SRS142781\_contig\_number\_650SRS142890\_contig\_number\_40387SRS142923\_contig\_number\_13709SRS142980\_contig\_number\_1215SRS143070\_contig\_number\_17148SRS143085\_contig\_number\_1178SRS143148\_contig\_number\_12836SRS143181\_contig\_number\_15932SRS143342\_contig\_number\_632SRS143372\_contig\_number\_5840SRS143417\_contig\_number\_19272SRS143523\_contig\_number\_16588SRS143722\_contig\_number\_11310SRS143780\_contig\_number\_2208SRS143876\_contig\_number\_11727SRS143895\_contig\_number\_31927SRS143991\_contig\_number\_24480SRS144135\_contig\_number\_36183SRS144183\_contig\_number\_41784SRS144297\_contig\_number\_1438SRS144362\_contig\_number\_24133SRS144537\_contig\_number\_49781SRS144603\_contig\_number\_1125SRS144714\_contig\_number\_22052SRS144753\_contig\_number\_2686SRS145308\_contig\_number\_516SRS145497\_contig\_number\_3392SRS146812\_contig\_number\_39714SRS146813\_contig\_number\_10095SRS147022\_contig\_number\_220SRS147039\_contig\_number\_contig-100\_1174.36190SRS147271\_contig\_number\_28898SRS147346\_contig\_number\_2135SRS147377\_contig\_number\_5501SRS147425\_contig\_number\_2192SRS147557\_contig\_number\_33920SRS147614\_contig\_number\_7169SRS147653\_contig\_number\_8230SRS147766\_contig\_number\_9425SRS147977\_contig\_number\_5910SRS148091\_contig\_number\_12211SRS148159\_contig\_number\_58262SRS148196\_contig\_number\_15244SRS148253\_contig\_number\_8078SRS148424\_contig\_number\_30277SRS148511\_contig\_number\_2882SRS148721\_contig\_number\_13194SRS148784\_contig\_number\_contig-100\_1235.152976SRS148817\_contig\_number\_12204SRS148970\_contig\_number\_1261SRS149075\_contig\_number\_12303SRS149181\_contig\_number\_29382SRS149244\_contig\_number\_2993SRS149784\_contig\_number\_17347SRS892882\_contig\_number\_15189SRS893170\_contig\_number\_505SRS893172\_contig\_number\_9424SRS893230\_contig\_number\_8972SRS893252\_contig\_number\_11593SRS893253\_contig\_number\_445SRS893256\_contig\_number\_1219SRS893259\_contig\_number\_2247SRS893270\_contig\_number\_22591SRS893279\_contig\_number\_4204SRS893285\_contig\_number\_1180SRS893288\_contig\_number\_629SRS893292\_contig\_number\_9503SRS893295\_contig\_number\_12699SRS893300\_contig\_number\_11805SRS893341\_contig\_number\_11763SRS893342\_contig\_number\_8821SRS893369\_contig\_number\_8003SRS893373\_contig\_number\_14871SRS893378\_contig\_number\_10392SRS893380\_contig\_number\_109SRS893382\_contig\_number\_1261SRS893383\_contig\_number\_417SRS971275\_contig\_number\_37040
species
821
351

species

SRS048981\_contig\_number\_5387
47678
1

162156
species

SRS047014\_contig\_number\_35764
1

2
817

SRS023829\_contig\_number\_556SRS1055099\_contig\_number\_10326
species

species

SRS054590\_contig\_number\_contig-100\_820.25858
310297
1


SRS013476\_contig\_number\_26458
species
457395
1


SRS150029\_contig\_number\_20837
species
29523
1


SRS144506\_contig\_number\_22685
species
2292934
1

1
28111
species

SRS893366\_contig\_number\_23505

2

SRS014923\_contig\_number\_29211SRS075021\_contig\_number\_6214
species
2292919

2292947
species

SRS049959\_contig\_number\_52386SRS077231\_contig\_number\_14929SRS098073\_contig\_number\_5910SRS149325\_contig\_number\_4078
4

246787

SRS014287\_contig\_number\_16975SRS893187\_contig\_number\_17891
species
2

2

SRS042966\_contig\_number\_6384SRS052027\_contig\_number\_6943
species
818

2
1263042

SRS1041038\_contig\_number\_16361SRS1055067\_contig\_number\_9801
species

2292935

SRS014613\_contig\_number\_3885SRS048060\_contig\_number\_12502SRS064232\_contig\_number\_2093SRS064276\_contig\_number\_contig-100\_87.224809SRS075963\_contig\_number\_contig-100\_3444.3444SRS098655\_contig\_number\_9293SRS1054691\_contig\_number\_5707SRS1055043\_contig\_number\_25091
species
8


SRS100021\_contig\_number\_3649SRS893358\_contig\_number\_9623
species
46506
2

family
171550
1

genus
239759
1

328814
species

SRS146764\_contig\_number\_18843
1
